# Surviving streptococcal toxic shock syndrome: a case report

**DOI:** 10.1186/1752-1947-1-118

**Published:** 2007-10-29

**Authors:** Thayur R Madhusudhan, Srivatsa Sambamurthy, Eileen Williams, Ian C Smith

**Affiliations:** 1 Registrar, Department of Orthopaedics, Glan Clwyd hospital, Rhyl, UK; 2 Registrar, Department of Anaesthesia, Glan Clwyd hospital, Rhyl, UK; 3 Consultant, Department of Anaesthesia, Glan Clwyd hospital, Rhyl, UK; 4 Consultant, Department of Orthopaedics, Glan Clwyd hospital, Rhyl, UK

## Abstract

Streptococcal toxic shock syndrome and associated myositis caused by group A beta-hemolytic streptococcus pyogenes generally have a poor outcome despite aggressive operative treatment. Frequently the diagnosis is missed initially as the clinical features are non-specific. The progression to a toxic state is rapid and unless definitive treatment measures are initiated early, the end result can be catastrophic. We report a previously healthy patient who had features of toxic shock syndrome due to alpha haemolytic (viridans) streptococcus mitis which was treated successfully with antibiotics, aggressive intensive care support including the use of a 'sepsis care bundle', monitoring and continuous multidisciplinary review. Life and limb threatening emergencies due to streptococcus mitis in an immune-competent person are rare and to our knowledge, have not previously been described in the English scientific literature. Successful outcome is possible provided a high degree of suspicion is maintained and the patient is intensively monitored.

## Case presentation

A previously healthy 33 year old woman was admitted to the acute medical care unit with a two day history of feeling lethargic and unwell. She was a non smoker and did not drink alcohol. She had developed severe continuous pain in the right upper limb with no specific aggravating or relieving factors and associated with severe weakness. She had had three episodes of vomiting and diarrhoea and had been treated by her general practitioner with paracetamol for flu-like symptoms for three days. On the morning of the day of admission she had noticed that her lips and tongue were swollen and she felt short of breath. She was given intramuscular adrenaline by the ambulance staff for a threatened airway and was transferred to the acute medical unit for further evaluation and management.

On admission she had pulse rate of 110/min, respiratory rate of 22/min and was febrile with a temperature of 38.9 deg c. She was agitated, restless and incontinent. Three hours later she started to develop swelling of the face. The facial skin was intact and there was no erythema, rash or blisters. Examination of the oral cavity revealed an oedematous tongue, pharynx and buccal mucosa with no detectable septic focus. There was no neck stiffness, no localising neurological signs and her GCS was 14. There was no local rise of temperature and soft tissue palpation of the head and neck was normal with no localised tenderness and no palpable lymph nodes or surgical emphysema. Air entry was equal on both sides but there were diffusely scattered rhonchi. Cardiovascular system examination was normal. Abdominal examination revealed a localised tenderness in the left iliac fossa. Pelvic and perineal examination by the gynaecologist was normal. Right upper limb examination was negative for any soft tissue or skeletal injuries. There were no signs of cellulitis, erythema or blisters. Palpation of the soft tissues was non tender, with no signs of grimacing or withdrawl and consistency was soft with no signs of compartment syndrome. Active movements at shoulder, elbow and wrist joints were within normal range and painless. All peripheral pulsations were normal with satisfactory capillary circulation.

The laboratory parameters following admission have been summarised in table 1. The C -reactive protein [CRP], urea, creatinine and leucocyte count were elevated. Arterial blood gas analysis was consistent with metabolic acidosis. Chest X-ray and CT scan of the abdomen were normal. Blood sample for Polymerised Chain Reaction [PCR] test for meningococcal antigen was negative. Lumbar puncture examination was normal. Synacthen stimulation test revealed a high baseline reading which was suggestive of maximal stimulation. A provisional diagnosis of meningococcal septicaemia was made and on microbiological advice treatment was commenced with a third generation cephalosporin and teicoplanin by intravenous route. Vital parameters were supported with intravenous fluids and oxygen therapy by mask. Intake output chart was strictly maintained.

**Table 1:** Haematology and biochemical parameters, from admission to discharge

Date	Day 1,	Day 2	Day 3	Day 3	Day 3	Day 4	Day 5	Day 5	Day 6	Day 7
**Time**	18.13 hrs	07 20 hrs	05 00 hrs	16 00 hrs	19 45 hrs	00 15 hrs	06 00 hrs	05 30 hrs	05 30 hrs	09 30 hrs

**Haemoglobin**	11.9	11.8	11.4	11	11.3	11.2	10.8	11.1	10.9	10.8
**Leucocyte count**	20.8	19.6	31.1	33.7	37.9	34.8	31.7	29.7	22.5	17.1
**Platelets**	151	130	90	90	94	86	86	108	156	281
**Haematocrit**	0.34	0.34	0.32	0.3	0.33	0.3	0.29	0.32	0.32	0.33
**Prothrombin time**	19	17.7	17.1	17.1	17.9	16.1	15.8	15.6	14.3	13.9
**APTT**	43.3	43.4	41	44.8	43.6	42.4	41.6	36.2	36	27.6
**Fibrinogen**	565	550	522	566	520	498	492	498	496	257
**Na**	132	138	134	134	134	134	135	137	143	136
**K**	3.1	4.1	4	3.9	3.9	3.8	3.4	4.1	4	3.6
**HCO3**	16	17	22	23	26	25	26	29	28	28
**Chlorides**	102	106	103	102	102	102	102	101	102	101
**Urea**	17.6	16.4	9.3	7.8	7.6	7	7.2	7.4	11.5	12
**Creatinine**	413	347	198	163	149	143	137	93	114	102
**Glucose**	9.3	8.5	7.7	7.2	7.4	7.4	7.4	5.7	6.6	6.5
**Albumin**	18	15	15	12	12	13	13	13	12	18
**CRP**	352	350	231	208	202	173	144	66	34.9	23.8
**Magnesium**	0.37	1.44	0.97	0.9	0.87	0.8	0.83	0.78	1.64	1.23
**Phosphorus**	0.5	1.1	0.8	0.6	1.2	0.7	0.8	0.9	1.1	1
**Calcium**	2.25	2.2	2.24	2.36	2.23	2.35	2.49	2.43	2.2	2.11
**Lactate**	3.7	1.9	1.4	1	1	1.1	1	1.2	1.1	1.1
**Creatinine kinase**	152	264	265	265	489	1428	3229	9367	5345	2123
**Bilirubin**	10	9	12	16	16	14	10	9	7	9
**ALT**	50	33	35	37	28	44	55	58	111	113
**AST**	31	34	35	67	77	79	104	295	197	204
**Globulin**	39	35	36	35	36	36	36	34	34	34
**Total proteins**	58	50	48	47	49	48	47	47	49	55

* All parameters in SI units

APTT – Activated partial thromboplastin time

ALT – Alanine transaminase

AST – Asparatate transaminase

CRP – C-reactive protein

HCO3 – Bicarbonate

Eight hours later her GCS dropped to 7 and in view of her threatened airway, she was transferred to the intensive care unit. She was further resuscitated, intubated, and ventilated. 'Sepsis care bundle' was commenced [1] which included optimisation of arterial pressure, haematocrit, central venous pressure and mixed venous saturation [2], low dose hydrocortisone administration following the synacthen test [3], intensive insulin therapy [4], and low tidal volume ventilation[5]. Renal replacement therapy by veno-venous filtration was begun because of the worsening acidosis. Recombinant human protein C was not given, as the criteria for its administration were not met [6].

**Figure 1:**
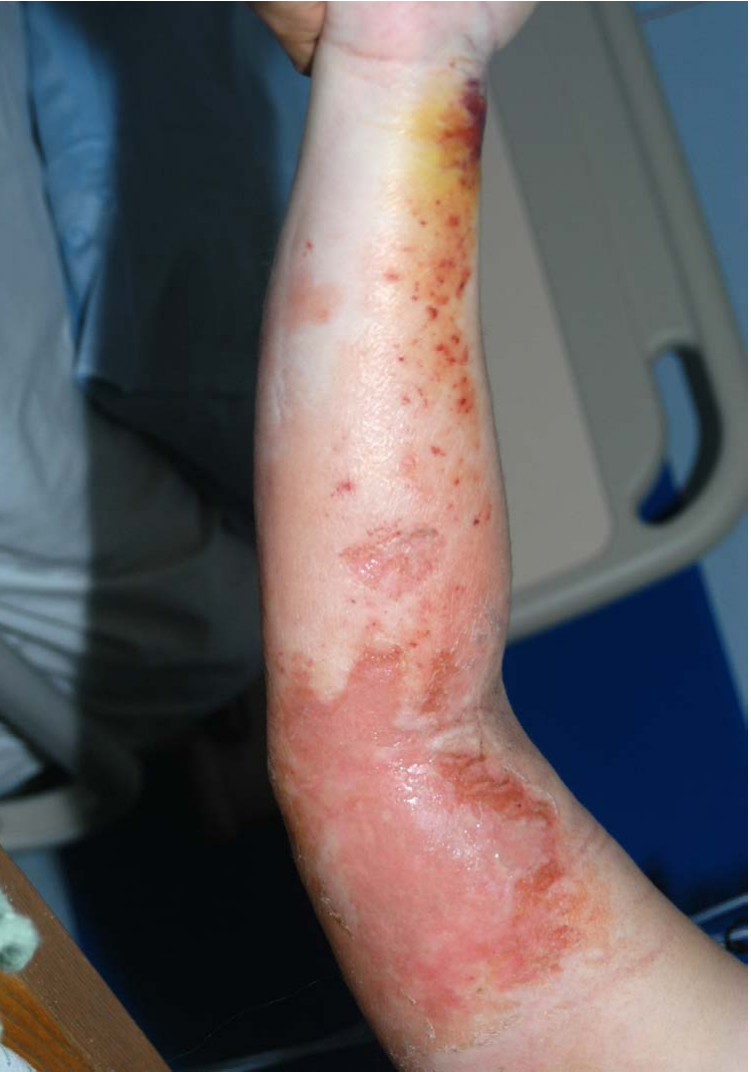
Day 3, post admission.

Over the next 18 hours a non-blanching erythematous rash appeared over the right upper limb, which progressed rapidly to a blister over the medial aspect of the arm with gross swelling. There was no local rise of temperature and the consistency was soft with satisfactory capillary circulation [Fig 1]. A diagnosis of necrotising fasciitis was considered. X-ray of the arm revealed no bony injury and no gas was evident in the subcutaneous tissues. The patient was hypotensive, clinically unstable and required constant monitoring and support. MRI was not available over the weekend and it was decided to proceed further on clinical assessment alone. Emergency surgery including the option of an amputation or a disarticulation was considered.

On microbiological advice, the antibiotics were changed to imipenem to cover both staphylococcal and streptococcal infection and clindamycin for its improved soft tissue penetration and ability to halt toxin production. Repeat blood samples revealed an elevated white cell count and CRP and normal coagulation parameters. Creatinine kinase was high and urine was negative for myoglobin. Initial blood cultures were negative and a repeat blood culture at 48 hours following admission was positive for alpha haemolytic (viridans) streptococcus mitis. The sepsis care bundle was continued, and the clinical and biochemical picture were reviewed frequently by senior orthopaedic, and intensive care staff.

**Figure 2:**
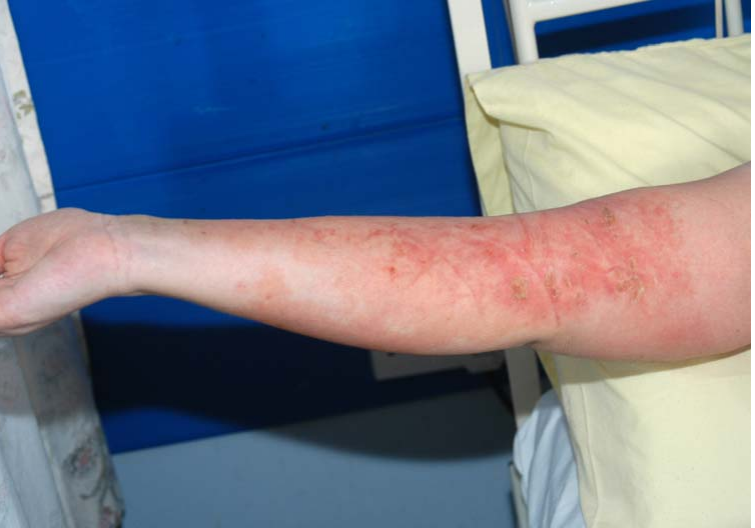
Day 10, post admission.

The patient improved within the next 20 hours and she was weaned from renal replacement therapy, ventilation and was extubated. She was transferred to the ward after 24 hours for further management. Oral clindamycin was continued for a further 2 weeks until her blood parameters were optimised. The skin lesions disappeared over the next three weeks with full recovery of her upper limb function [Fig 2].

## Discussion

Streptococcal toxic shock syndrome is a distinct entity, which is aggressive compared to the milder forms of streptococcal infections such as pyomyositis and fasciitis [7]. Earlier cases of streptococcal myositis described in the literature indicate that the causative organism is streptococcus pyogenes [7-14] and the condition presents with a uniform pattern of involvement, with the lower limbs more commonly involved [8,9]. A high index of suspicion at the onset may present the only chance of survival in these infections as the disease has mortality as high as 85 % [10,11]. This poor outcome is due in large part to the non-specific features at presentation, leading to misdiagnosis or delayed diagnosis, the aggressive nature of infection which is frequently underestimated, and delay in appropriate administration of antibiotics and surgical debridement [12]. Although many specific variations of necrotising soft tissue infections have been described on the basis of etiology, microbiology, and specific anatomic location of the infection, the initial approach to the diagnosis, antimicrobial treatment, and decision to use operative management are similar for all forms and are more important than determining the specific variant. [15]

The guidelines for management of severe sepsis and septic shock have been developed [1]. In our patient a definitive diagnosis could not be made until 34 hours after admission. The possible reasons for this delay could be the vague history and non-specific clinical features, the absence of a clinically detectable septic focus in a previously healthy patient and the time required for organisms to be cultured in the laboratory. The patient was screened for all the possible pathogens from vulnerable areas including an MRSA screening and only the second blood culture sample sent from the Intensive Care Unit was reported positive for streptococcus mitis. The microbiologist was unsure of the clinical significance because of the normal commensal behaviour of the organism. Nevertheless clindamycin therapy was initiated in addition to teicoplanin. This highlights the importance of a persistent diligent search for a possible focus with repeated clinical examination and laboratory investigations in clinically vague situations. The early institution of goal directed therapy with sepsis care bundle probably led to a reduction in the bacterial toxin load and may have lessened the degree of end organ damage. Nevertheless the patient had impaired renal and hepatic function for seven days in the recovery period.

When an orthopaedic opinion was sought the patient was toxaemic and was on ventilatory support. A distinguishing clinical feature of necrotising fasciitis is the wood-hard feel of the subcutaneous tissues. The underlying tissues are firm, and the fascial planes and muscle groups cannot be discerned by palpation. It is often possible to observe a broad erythematous tract in the skin along the route of the infection as it advances cephalad in an extremity. If there is an open wound, probing the edges with a blunt instrument permits ready dissection of the superficial fascial planes well beyond the wound margins [15]. In our patient, the hard wooden feeling of the soft tissues was characteristically absent. A positive blood culture, serial high levels of creatinine kinase, and a negative urine test for myoglobin along with the clinical features were highly indicative for streptococcal myositis. MRI though useful may not be possible in an unstable patient or may be unavailable for several reasons. Further, data regarding the sensitivity and specificity of CT or MRI are unavailable, and requesting such studies may delay definitive diagnosis and treatment. In practice, clinical judgment is the most important element in diagnosis [15].

The diagnosis of compartment syndrome requires a high degree of clinical judgement and is difficult in a ventilated patient. Monitoring of pressure has no harmful effect and may allow early fasciotomy, although the intracompartmental pressure threshold for such an undertaking is still unclear [16]

The decision to undertake aggressive surgery should be based on several considerations the commonest being failure to respond to antimicrobial therapy [15]. In our patient the option of a vigorous debridement including amputation or a disarticulation was considered initially. However on balance, the decision to operate was deferred as there was clinical improvement in the next 12 hours. A definitive tissue diagnosis of streptococcal mitis was therefore not possible.

Streptococcus mitis is an alpha haemolytic streptococcus of the viridans group, normally a commensal in the oral cavity and is known to cause endocarditis in patients with damaged heart valves. It lacks the polysaccharide capsule and antigenic protein which is typically present in the pyogenic strains of streptococci [17,18]. Streptococcal toxic shock syndrome has been reported to occur in immunocompromised hosts with bone marrow transplants due to alpha haemolytic (viridans) streptococcus mitis [19].

Streptococcal toxic-shock syndrome is defined as any group A streptococcal infection associated with the early onset of shock and organ failure. The criteria for diagnosis have been well described and are summarised in Table 2[20].

**Table 2:** Streptococcal Toxic Shock Syndrome

	1	2
**Criterion A**	Sterile site	Non sterile body site
**Criterion B**	Hypotension	Clinical and laboratory abnormalities including renal impairment, Liver abnormalities, Acute respiratory distress syndrome, extensive tissue necrosis ie. Necrotising fascitis and erythematous rash **(requires two or more for diagnosis)**

Criterion A = Isolation of group A Streptococcus

Criterion B = Clinical signs of severity

Definite Case = A1 + B (1+2)

Probable Case = A2 + B (1+2)

Streptococcal toxic shock syndrome caused by group A streptococci should be treated with clindamycin and penicillin. The rationale for clindamycin is based on in vitro studies demonstrating both toxin suppression and modulation of cytokine (i.e. TNF) production, on animal studies demonstrating superior efficacy compared to penicillin, and on two observational studies demonstrating greater efficacy for clindamycin than for β-lactam antibiotics [20,21]. It has also been suggested that in vitro sensitivity of group A haemolytic streptococci to penicillin may not be effective in vivo [22] and clindamycin may be more efficacious but has declining efficacy if treatment is delayed [22,23].

Anecdotal evidence suggests that intravenous immunoglobulin [IVIG] may have a place in neutralising the secreted streptococcal toxins that are thought to mediate features of the disease [24]. The use of a medical regimen including IVIG in patients with severe Group A Streptococcal soft tissue infections may allow an initial non-operative or minimally invasive approach which can limit the need to perform immediate wide debridements and amputations in unstable patients [25]. However a recommendation on the use of IVIG to treat streptococcal toxic shock syndrome cannot be made with certainty. Although there is ample evidence for the role of extracellular streptococcal toxins in shock, organ failure, and tissue destruction, different batches of IVIG contain variable quantities of neutralising antibodies to some of these toxins and definitive clinical data are lacking [26].

Other case reports of streptococcal toxic shock syndrome with streptococcus pyogenes report high patient mortality despite aggressive surgical debridement. Early diagnosis and aggressive supportive treatment may alter the final outcome favourably [8,11,12]. This probably is true and as in our patient may result in better outcomes with streptococcus mitis infections.

## Conclusion

Streptococcal toxic shock syndrome due to alpha-haemolytic [viridians] streptococcus mitis infection in a previously healthy person is possible and can present as a life or limb-threatening emergency. This needs early recognition and successful outcome is possible by maintaining a high index of suspicion, early diagnosis and aggressive organ support.

## Authors' contributions

Mr T R Madhusudhan (Author 1) and Mr I C Smith (Author 4) were actively involved in the management and decision making from the orthopaedic team while the patient was in the ICU and ward until final discharge and at follow up.

Dr Srivatsa Sambamurthy (Author 2) and Dr Eileen Williams (Author 3) were actively involved from the anaesthetic team, in the management of the patient including resuscitation, treatment and optimisation of the patient following admission to the ICU.

All the above authors have read and approved the final manuscript.
